# Supplementary material for: Post-Harvest Processing Methods Have Critical Roles in the Contents of Active Ingredients of Scutellaria baicalensis Georgi
Source: Molecules. 2022 Nov 28;27(23):8302. doi: 10.3390/molecules27238302 (PMC9739255; doi:10.3390/molecules27238302)
Supplement: Supplementary file 1 [file molecules-27-08302-s001.zip › molecules-2007821-supplementary.pdf]

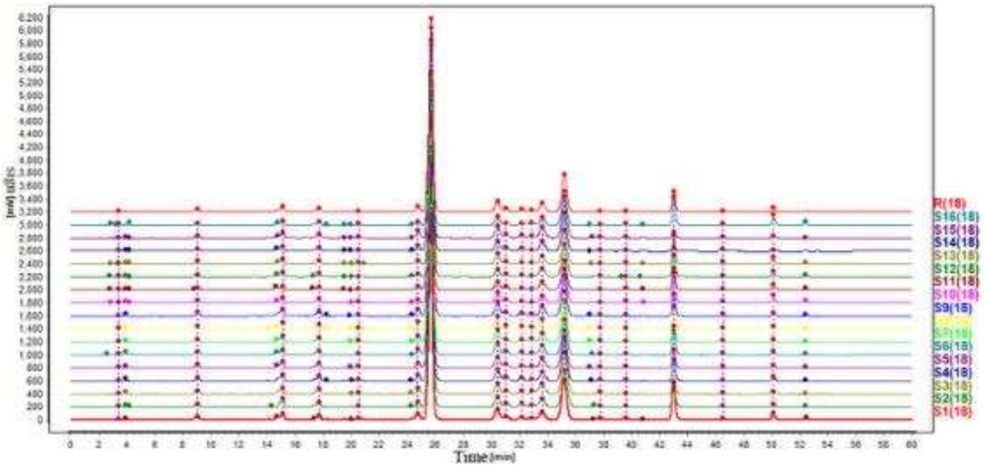

Figure S1. Fingerprints of *S.baicalensis* in 16 batches of different processing processes

Table S1. The relative retention time of the common peaks of the 16 batches of *S. baicalensis* with different processing

NO.	The Relative Retention Time																RSD/ %
	S1	S2	S3	S4	S5	S6	S7	S8	S9	S10	S11	S12	S13	S14	S15	S16	
1	0.096	0.098	0.096	0.096	0.098	0.098	0.097	0.097	0.097	0.096	0.097	0.096	0.097	0.098	0.096	0.098	0.744
2	0.258	0.262	0.257	0.264	0.260	0.265	0.260	0.260	0.261	0.258	0.260	0.259	0.260	0.249	0.258	0.262	1.352
3	0.431	0.436	0.428	0.441	0.433	0.442	0.433	0.433	0.435	0.431	0.430	0.431	0.432	0.429	0.431	0.435	0.891
4	0.504	0.510	0.500	0.514	0.506	0.516	0.506	0.506	0.508	0.505	0.504	0.504	0.506	0.506	0.505	0.508	0.788
5	0.583	0.591	0.577	0.577	0.586	0.598	0.586	0.587	0.582	0.583	0.583	0.582	0.585	0.584	0.584	0.590	0.865
6	0.704	0.704	0.689	0.696	0.704	0.723	0.704	0.705	0.703	0.704	0.703	0.694	0.704	0.705	0.704	0.702	0.995
7	0.731	0.730	0.704	0.710	0.730	0.743	0.729	0.731	0.728	0.730	0.729	0.735	0.730	0.730	0.731	0.728	1.249
8	0.865	0.866	0.866	0.853	0.864	0.878	0.864	0.864	0.863	0.864	0.864	0.864	0.864	0.863	0.864	0.864	0.526
9	0.884	0.885	0.885	0.871	0.881	0.909	0.881	0.881	0.880	0.882	0.882	0.883	0.882	0.882	0.882	0.880	0.856
10	0.915	0.913	0.901	0.889	0.913	0.931	0.913	0.914	0.913	0.913	0.913	0.913	0.913	0.913	0.913	0.913	0.915
11	0.937	0.931	0.914	0.917	0.932	0.951	0.932	0.932	0.931	0.935	0.935	0.939	0.934	0.929	0.935	0.931	0.875
12	0.958	0.953	0.942	0.936	0.954	0.973	0.955	0.955	0.955	0.955	0.956	0.957	0.955	0.954	0.956	0.954	0.788
13	1.000	1.000	1.000	1.000	1.000	1.000	1.000	1.000	1.000	1.000	1.000	1.000	1.000	1.000	1.000	1.000	0.000
14	1.071	1.066	1.060	1.052	1.077	1.077	1.077	1.077	1.077	1.072	1.071	1.067	1.073	1.070	1.072	1.078	0.659
15	1.120	1.117	1.120	1.118	1.126	1.134	1.125	1.126	1.127	1.122	1.121	1.115	1.124	1.123	1.123	1.127	0.422
16	1.219	1.218	1.208	1.214	1.225	1.240	1.224	1.224	1.226	1.222	1.219	1.216	1.223	1.222	1.220	1.225	0.559
17	1.318	1.320	1.306	1.310	1.325	1.345	1.322	1.324	1.326	1.321	1.318	1.315	1.323	1.321	1.319	1.325	0.632
18	1.419	1.427	1.406	1.410	1.428	1.449	1.425	1.421	1.429	1.422	1.419	1.416	1.424	1.422	1.421	1.429	0.658

Table S2. The similarity results of the common peaks of the 16 batches of *S. baicalensis* with different processing.

NO.	S1	S2	S3	S4	S5	S6	S7	S8	S9	S10	S11	S12	S13	S14	S15	S16	Sr.
**S1**	1.000	0.997	0.994	0.998	0.996	0.998	0.996	0.997	0.997	1.000	0.998	0.998	0.999	0.990	0.992	0.999	0.998
**S2**	0.997	1.000	0.999	1.000	1.000	1.000	1.000	1.000	1.000	0.998	0.999	0.999	0.998	0.997	0.998	0.998	1.000
**S3**	0.994	0.999	1.000	0.999	1.000	0.999	1.000	0.999	0.999	0.995	0.998	0.999	0.996	0.999	0.999	0.996	0.999
**S4**	0.998	1.000	0.999	1.000	1.000	1.000	1.000	1.000	1.000	0.998	0.999	0.999	0.999	0.997	0.998	0.998	1.000
**S5**	0.996	1.000	1.000	1.000	1.000	1.000	1.000	1.000	1.000	0.997	0.999	0.999	0.998	0.998	0.999	0.998	1.000
**S6**	0.998	1.000	0.999	1.000	1.000	1.000	0.999	1.000	1.000	0.999	0.999	1.000	0.999	0.996	0.997	0.999	1.000
**S7**	0.996	1.000	1.000	1.000	1.000	0.999	1.000	0.999	0.999	0.997	0.998	0.998	0.997	0.998	0.999	0.997	0.999
**S8**	0.997	1.000	0.999	1.000	1.000	1.000	0.999	1.000	1.000	0.998	1.000	1.000	0.999	0.997	0.998	0.998	1.000
**S9**	0.997	1.000	0.999	1.000	1.000	1.000	0.999	1.000	1.000	0.998	0.999	0.999	0.999	0.997	0.998	0.998	1.000
**S10**	1.000	0.998	0.995	0.998	0.997	0.999	0.997	0.998	0.998	1.000	0.998	0.999	1.000	0.991	0.993	1.000	0.998
**S11**	0.998	0.999	0.998	0.999	0.999	0.999	0.998	1.000	0.999	0.998	1.000	1.000	0.999	0.996	0.997	0.999	0.999
**S12**	0.998	0.999	0.999	0.999	0.999	1.000	0.998	1.000	0.999	0.999	1.000	1.000	0.999	0.996	0.997	0.999	1.000
**S13**	0.999	0.998	0.996	0.999	0.998	0.999	0.997	0.999	0.999	1.000	0.999	0.999	1.000	0.993	0.994	1.000	0.999
**S14**	0.990	0.997	0.999	0.997	0.998	0.996	0.998	0.997	0.997	0.991	0.996	0.996	0.993	1.000	1.000	0.992	0.997
**S15**	0.992	0.998	0.999	0.998	0.999	0.997	0.999	0.998	0.998	0.993	0.997	0.997	0.994	1.000	1.000	0.994	0.998
**S16**	0.999	0.998	0.996	0.998	0.998	0.999	0.997	0.998	0.998	1.000	0.999	0.999	1.000	0.992	0.994	1.000	0.999
**Sr.**	0.998	1.000	0.999	1.000	1.000	1.000	0.999	1.000	1.000	0.998	0.999	1.000	0.999	0.997	0.998	0.999	1.000

Table S3. The relative peak area of the common peaks of the 16 batches of *S. baicalensis* with different processing.

NO.	The Relative Peak Area																RSD/%
	S1	S2	S3	S4	S5	S6	S7	S8	S9	S10	S11	S12	S13	S14	S15	S16	
1	0.009	0.009	0.010	0.008	0.009	0.017	0.008	0.008	0.008	0.015	0.016	0.016	0.009	0.009	0.006	0.017	35.487
2	0.063	0.060	0.065	0.065	0.068	0.068	0.053	0.063	0.067	0.065	0.047	0.055	0.075	0.049	0.045	0.042	16.242
3	0.098	0.082	0.146	0.146	0.113	0.164	0.099	0.109	0.167	0.101	0.092	0.106	0.111	0.088	0.102	0.106	23.091
4	0.082	0.083	0.098	0.102	0.103	0.103	0.097	0.110	0.111	0.101	0.072	0.080	0.102	0.104	0.093	0.097	11.813
5	0.029	0.029	0.019	0.014	0.032	0.032	0.026	0.023	0.023	0.029	0.023	0.023	0.032	0.020	0.022	0.030	21.160
6	0.147	0.082	0.149	0.014	0.082	0.082	0.098	0.132	0.110	0.074	0.118	0.023	0.100	0.092	0.137	0.084	40.491
7	3.993	3.966	0.089	0.100	4.230	4.233	3.852	4.436	4.248	4.178	4.859	4.505	4.348	4.501	4.300	4.167	38.584
8	0.261	0.249	0.249	0.241	0.272	0.088	0.268	0.312	0.310	0.303	0.283	0.232	0.294	0.248	0.285	0.249	20.195
9	0.082	0.087	0.247	0.276	0.093	0.077	0.062	0.089	0.080	0.089	0.124	0.085	0.076	0.080	0.097	0.057	58.953
10	0.070	0.074	0.086	0.093	0.067	0.056	0.061	0.079	0.090	0.065	0.072	0.077	0.068	0.091	0.080	0.061	15.424
11	0.054	0.058	0.058	0.068	0.062	0.255	0.081	0.052	0.058	0.032	0.054	0.045	0.057	0.048	0.058	0.072	73.144
12	0.216	0.255	0.072	0.046	0.286	0.286	0.217	0.270	0.275	0.237	0.306	0.293	0.249	0.337	0.306	0.255	32.540
13	1.000	1.000	1.000	1.000	1.000	1.000	1.000	1.000	1.000	1.000	1.000	1.000	1.000	1.000	1.000	1.000	0.000
14	0.035	0.029	0.029	0.018	0.027	0.039	0.032	0.030	0.034	0.033	0.036	0.050	0.034	0.025	0.035	0.034	21.316
15	0.042	0.032	0.032	0.030	0.041	0.035	0.022	0.030	0.030	0.059	0.054	0.021	0.045	0.018	0.032	0.039	32.273
16	0.623	0.337	0.214	0.351	0.305	0.430	0.249	0.392	0.356	0.599	0.487	0.445	0.577	0.058	0.104	0.561	44.518
17	0.022	0.017	0.026	0.018	0.026	0.032	0.024	0.024	0.028	0.027	0.027	0.019	0.042	0.028	0.025	0.032	23.584

18	0.108	0.048	0.034	0.063	0.039	0.060	0.037	0.046	0.049	0.131	0.104	0.097	0.118	0.017	0.044	0.179	60.413

Table S4. Principal components initial eigenvalue and contribution rate

Principal Components	Eigenvalues	Contribution Rate (%)	Cumulative Contribution Rate(%)
1	4.563	32.59	32.59
2	3.242	23.16	55.75
3	2.304	16.455	72.205
4	1.476	10.541	82.747

Table S5. Factor loading matrix after rotation.

	Principal Components			
**Peak no.**	**1**	**2**	**3**	**4**
A3	0.625	0.03	0.655	0.137
A4	0.764	0.343	0.218	0.415
A7	-0.417	0.846	-0.24	0.129
A8	0.367	0.018	-0.694	0.518
A9	0.646	-0.718	0.155	-0.125
A10	0.889	-0.037	-0.263	0.009
A11	0.068	0.36	0.87	-0.183
A12	-0.212	0.906	-0.245	0.009
A13	0.807	0.344	0.068	0.119
A14	-0.327	0.563	0.216	-0.355
A15	-0.483	-0.332	0.158	0.591
A16	-0.653	-0.3	0.32	0.325
A17	0.018	0.428	0.427	0.55
A18	-0.778	-0.349	0.135	0.227

1. Validation of methodology

According to the Pharmacopeia guidelines of the People's Republic of methodology was performed with linear regression analysis and tests of precision, repeatability, stability, and recovery rate.

1.1. Linear relationships

Each standard curve was obtained by plotting the PA (y) against the mixed standard solution concentrations (x, $\mu g \cdot ml^{-1}$). The results are shown in Table S6. The four ingredients each had an excellent linear relationship within their respective detection ranges and the linear coefficient of determination (r2) exceeded 0.999.

1.2. Precision, repeatability, stability, and recovery tests

Method precision and repeatability were evaluated via successive analyses of four replicates of the same powder sample. The relative standard deviation (R.S.D.) was 0.06-1.50% for retention time (tR) and 0.41-2.17% for the PA of four characteristic peaks.

These findings showed that the method had good repeatability and precision. The stab ility of the sample solutions was determined by analyzing samples at 0, 2, 4, 8, 16, an d 24 h after solution preparation. The R.S.D. was 0.23-1.30% for tR and 0.73-2.33% for the PA of the characteristic peaks. These results showed that the sample solution had good stability within 24 h. The recovery rate was determined by the standard addition method. The recovery of the four standards ranged from 96.92% to 104.83%. The R.S. D. was 0.96-3.52% for PA. The results of the precision, repeatability, stability and reco very tests are shown in Table S7 and Table S8.

Table S6. Linear relationships table of four active ingredients.

Components	Regression Equation	R^2	Linear Range ($ug{\cdot}mL^{-1}$)
Baicalin	y = 62,856,944.1952 x - 392,012.1028	0.9998	6.58～657.50
Wogonoside	y = 72,138,920.7031 x - 19,176.7275	0.9997	1.00～100.00
Baicalein	y = 43,555,907.2718 x - 82,035.0058	0.9997	28.72～2871.80
Wogonin	y = 52,715,929.0570 x - 467,342.8039	0.9996	10.00～1000.00

Table S7. Analytical results of precision, and stability of four ingredients.

Components	Precision (n=6)		Stability (n=6)	
	RSD of relative Retention time (%)	RSD of relative Retention Area (%)	RSD of relative Retention time (%)	RSD of relative Retention Area (%)
Baicalin	1.50	1.50	1.28	2.24
Wogonoside	0.40	0.53	1.30	0.73
Baicalein	0.12	0.41	0.76	0.89
Wogonin	0.06	1.72	0.23	2.33

Table S8. Analytical results of repeatability and recovery tests of four ingredients

Components	Repeatability (n = 6)		Recovery rate (n = 6)	
	RSD of Relative Retention Time (%)	RSD of Relative Retention Area (%)	Recovery Rate (%)	RSD of Retention Area (%)
Baicalin	0.68	2.17	104.83	2.34
Wogonoside	0.90	1.99	102.66	0.96
Baicalein	0.27	1.54	96.92	3.52
Wogonin	0.19	1.72	100.38	1.71
